# Review on the Clinical, Imaging, and Therapeutic Aspects of Cardiac Masses in Dog

**DOI:** 10.3390/life15071092

**Published:** 2025-07-11

**Authors:** Nicoleta Andreea Mincă, Ionuț Cătălin Dumbravă, Niculae Tudor, Alina Ștefănescu, Alexandru Bogdan Vițălaru, Lucian Ioniță, Dorin Țogoe

**Affiliations:** Faculty of Veterinary Medicine, University of Agronomic Sciences and Veterinary Medicine of Bucharest, 59 Marasti Blvd, District 1, 011464 Bucharest, Romania; nicoleta.minca@fmvb.usamv.ro (N.A.M.); ktalin13@yahoo.com (I.C.D.); niculae.tudor@fmvb.usamv.ro (N.T.); alina.stefanescu@fmvb.usamv.ro (A.Ș.); alexandru.vitalaru@fmvb.usamv.ro (A.B.V.); lucian.ionita@fmvb.usamv.ro (L.I.)

**Keywords:** cardiac tumors, hemangiosarcoma, chemodectoma, tumor features

## Abstract

Cardiac tumors in dogs, although rare in the past, have shown an increasing incidence due to advances in veterinary imaging, especially echocardiography, CT, and MRI with contrast agents. Right atrial hemangiosarcoma is the most common form of tumor associated with pericardial effusion and cardiac tamponade, followed by chemodectoma, which is more common in brachycephalic breeds. The diagnosis is based on echocardiographic examination, supplemented by advanced methods and possibly biopsy. Clinical signs are often non-specific, requiring an integrated approach. Treatment includes pericardiocentesis, chemotherapy, and, in some cases, surgery, but the prognosis remains reserved.

## 1. Introduction

In recent years, cardiac masses in canine species have attracted growing interest from veterinary oncology. Indeed, although considered rare in veterinary medicine, they have shown a notable increase in incidence. This trend is also driven by the continuous advancement of diagnostic imaging techniques, as well as severe evolutionary potential and the often clinically dramatic manifestations of these lesions. In this context, the identification and classification of cardiac tumor masses have seen an increasing incidence and innovative treatment methods with them [1].

Cardiac neoplasms in canids have been described as primary or secondary masses, the best-known neoplasms being hemangiosarcoma, chemodectoma, ectopic thyroid carcinoma, lymphoma, and myxoma [2]. Some authors also add pericardial mesothelioma to this category, although it is not considered a primary cardiac tumor but a pericardial tumor with cardiological implications [3]. In dogs, hemangiosarcoma is the most common type of heart tumor, followed by chemodectoma and myocardial lymphoma [4].

The symptomatology is often non-specific, in some cases even absent, and is most often associated with heart failure or respiratory failure, vascular collapse, hemothorax, hemopericardium, or ascites [5].

Imaging plays an essential role in the diagnosis of these pathologies. Echocardiography is the main evaluation method with a sensitivity between 16.7% and 80% [6], providing valuable information on location and size. Advanced imaging methods like CT and MRI are frequently employed to supplement echocardiography when atypical or diffuse infiltrative patterns are present. Although they offer accurate visualization and staging, they involve higher anesthesia risks in patients with heart disease [7].

Therapeutic management, including palliative care, surgery, and oncology, has greatly improved in recent years, increasing survival rates. It is our view that the ongoing advancement and diversification of veterinary imaging diagnostic techniques have contributed to increased detection of these cardiac structures. At present, cardiac tumors are not considered to be associated with environmental or genetic predisposition factors.

## 2. Relevant Sections

In the past, primary cardiac neoplasia has been rarely reported, but it is seeing an increasing incidence, with 0.12% reported in 1965 [8], 0.17% reported in 1995 [9], 0.19% reported in 1999 [10], 0.17–0.19% reported in 2013 [11], 2.57% reported in 2016 [12], and 0.17–0.19% reported in 2018 [13]. It is currently considered to be at an optimal range reported between 0.12% and 0.19% in canids [12]. Cardiac tumors can be classified based on their origin, histological characteristics (benign or malignant), echocardiographic appearance, or macroscopic features, such as size, shape, location, and attachment site. Primary cardiac tumors are more frequently observed in large, middle-aged dog breeds with no apparent gender predisposition. An exception is lymphoma, which tends to occur in dogs older than seven years of age. A predisposition for certain tumors has been reported; for example, chemodectoma is more common in brachycephalic breeds, while atrial hemangiosarcoma is more frequently identified in large breeds such as the German Shepherd, Labrador Retriever, and Golden Retriever [14].

Less common heart tumors include neoplasms such as osteosarcomas, fibrosarcomas, fibromas, cardiac lipomas, malignant mesenchymal tumors, and rhabdomyosarcomas [15].

Diagnosis is frequently based on the echocardiographic localization of the lesion, echocardiographic texture, and structure extension [6,16].

## 3. Discussion

### 3.1. Clinical Examination

Anamnesis is a critical component in diagnosing cardiovascular diseases. When performed thoroughly, it can guide the clinician toward a cardiovascular etiology. The clinical examination begins with the palpation of the cardiac area, which helps identify the point of maximal impulse (PMI) and assess its intensity and location.

Auscultation remains a fundamental technique in the clinical evaluation of cardiac function. It involves the assessment and interpretation of heart sounds. In dogs with cardiac tumors, auscultatory findings are often non-specific, depending on the tumor’s location, size, and related complications such as pericardial effusion [1,5]. The data are shown in Table 1.

The most commonly observed abnormality is muffled heart sounds, typically due to pericardial effusion or cardiac tamponade, frequently associated with hemangiosarcoma, chemodectoma, mesothelioma, and lymphoma [1,5]. When present, heart murmurs are generally low-grade (I–III/VI) and may result from altered intracardiac blood flow, mild valvular involvement, or chamber obstruction [14]. Tumors such as ectopic thyroid neoplasms or myxosarcomas may only produce murmurs if they interfere with valvular function or outflow tracts [17].

Additional auscultatory findings such as gallop rhythms or extra heart sounds can occur when restrictive physiology or right-sided heart failure develops, particularly in cases involving extensive pericardial disease, as seen in mesothelioma and sarcomas [14]. While auscultation provides valuable information, it is limited in its ability to detect cardiac masses and should always be interpreted in conjunction with imaging methods, particularly echocardiography [18].

Key clinical signs that may suggest cardiovascular disease include dyspnea (either progressive or persistent) and coughing, which may result from pulmonary congestion or the compression of adjacent structures [19].

Chemodectomas, commonly located at the base of the heart, often remain asymptomatic until they enlarge sufficiently enough to compress surrounding tissues. These tumors are associated with hypoxia and are more frequently observed in brachycephalic breeds [20].

Cardiac hemangiosarcomas may obstruct blood flow or rupture vessels, leading to pericardial effusion and, ultimately, cardiac tamponade. These tumors often induce right-sided heart failure, with clinical signs that may include ascites, jugular venous distension, and abnormal jugular pulsations. Additional symptoms such as lethargy and exercise-induced syncope are also common. Hemopericardium caused by the rupture of the right atrial wall is a hallmark of cardiac hemangiosarcoma [21].

These signs often result from tamponade, vascular obstruction, arrhythmias, or structural and functional myocardial damage [21]. In most cases, the presence of pericardial effusion is the primary determinant of clinical manifestations [1].

Initial, general symptoms such as reduced activity, inappetence, exercise intolerance, dyspnea, mucosal pallor, weight loss, and episodic collapse are non-pathognomonic and usually require further diagnostic evaluation through imaging and laboratory testing. Malignant cardiac tumors, particularly cardiac hemangiosarcomas, are a common cause of cardiac tamponade, a life-threatening condition caused by the accumulation of fluid in the pericardial sac, which restricts diastolic filling and significantly reduces cardiac output. The severity of clinical signs depends on both the volume and the rate of pericardial fluid accumulation. Early signs, such as lethargy, inappetence, and exercise intolerance, typically precede the development of ascites, especially in acute effusions. Frequently observed clinical signs include tachypnea, progressive abdominal distension, generalized weakness, syncope or collapse, and occasional coughing. Vomiting may also occur and can reflect systemic hypoperfusion, which is often correlated with elevated plasma lactate levels. On physical examination, affected dogs may present jugular vein distension, positive hepatojugular reflux, hepatomegaly, abdominal effusion, labored breathing, and weak femoral pulses. Common signs of poor perfusion include sinus tachycardia, pale mucous membranes, and a prolonged capillary refill time. Pulsus paradoxus may also be detected through the careful palpation of the femoral pulse. Notably, cardiac hemangiosarcoma is the most common cause of hemopericardium, frequently resulting from the rupture of the right atrial wall, and is strongly associated with right-sided heart failure, evident through ascites, persistent jugular distension and abnormal jugular pulsations, as well as exertional collapse and syncope due to obstructed blood flow and myocardial compromise [22]. The data are shown in Table 2.

Cardiomegaly, reflected by an increased Vertebral Heart Score (VHS), may lead to airway compression, resulting in coughing as a clinical symptom. Additionally, right-sided congestive heart failure can manifest with gastrointestinal signs, such as diarrhea [23,24,25].

Cardiac biomarkers, including the N-terminal pro-B-type natriuretic peptide (NT-proBNP) and cardiac troponin I (cTnI), may serve as adjunctive laboratory tests in the diagnostic evaluation of cardiac tumors in dogs. NT-proBNP reflects myocardial wall stress and volume overload, and its concentration may rise in the presence of tumors that disrupt normal cardiac hemodynamics. Likewise, cTnI is a highly sensitive marker for myocardial cell injury and may be elevated in cases involving infiltrative or invasive cardiac neoplasms. Despite their clinical utility, both markers are limited by a lack of specificity, as elevated levels can also be observed in various non-neoplastic cardiac disorders.

#### Diagnostic Imaging Techniques

The increase in the incidence of cardiac masses diagnosed in veterinary medicine can be directly attributed to the progress of imagistic investigation methods, especially echocardiography [7]. In veterinary medicine, the evaluation of cardiac tumors in dogs using advanced imagistic techniques often involves the use of contrast agents to highlight tumor masses and adjacent structures [26]. In computed tomography (CT), iodine-based contrast agents are used, which can be divided into ionic monomers with high osmolarity and a higher prevalence of side effects and nonionic monomers with low osmolarity, which are better tolerated and frequently used in veterinary practice [27]. The administration protocol for CT angiography in dogs includes an iodine concentration of 300–400 mg/mL, injected at a rate of 4–6 mL/s, in a volume of approximately 1–2 mL/kg, followed by a saline flush, using synchronization techniques such as the bolus test to optimize the timing of the scan [7]. In magnetic resonance imaging (MRI), gadolinium-based contrast agents—either linear or macrocyclic—are essential for differentiating tumor tissues from normal myocardium. It is administered in doses of 0.05–0.1 mmol/kg for myocardial infusion and 0.1–0.2 mmol/kg for the delayed assessment, with an injection rate of 3–7 mL/s, followed by washing with 30 mL of saline [26]. Contrast infusion imaging can help differentiate the tumor mass from the adjacent myocardium: malignant tumors and those with intense vascularization are hyperintense, while stromal tumors and thrombi appear hypointens [7]. These protocols allow for a more precise characterization of cardiac tumors and can guide therapeutic decisions in canine patients, but the administration of contrast agents in cardiac imaging techniques in dogs involves the potential risk of adverse reactions. The most common are hypersensitivity reactions, which can range from mild forms (pruritus, erythema, urticaria) to severe reactions, such as angioedema, bronchospasm, or cardiovascular collapse, especially in patients with congestive heart failure [27]. Also, administering a large volume of fluid quickly can lead to volume overload and acute pulmonary edema, especially in dogs with low ejection fraction, valvulopathies, or restrictive cardiomyopathies. Contrast agents can influence myocardial excitability, favoring the appearance of arrhythmias, especially in the context of pharmacological manipulations such as the administration of beta-blockers, which are used to control the heart rhythm in CT or MRI. In addition, dogs with pre-existing renal dysfunction and cardiovascular conditions are prone to contrast-induced nephropathy (CIN), a complication with a negative impact on cardio-renal status and overall prognosis [7].

For diagnosis, echocardiography has proven to be an essential non-invasive diagnostic technique method, although it has some limitations in the detection of infiltrative tumors. Increasingly, these types of tumors are identified incidentally during routine evaluations, especially in the context of non-specific changes in the patient’s general condition (such as lethargy, apathy, or anorexia) or in the presence of pericardial effusions. Pericardial effusion is often a major clinical alarm signal and is strongly associated with the presence of cardiac neoplasms, especially in the case of hemangiosarcoma. Echocardiography, although it does not provide histological certainty, remains the main non-invasive diagnostic tool, with a sensitivity of up to 80% in detecting cardiac masses [28]. This allows the location, size, and relationship with adjacent structures to be assessed. However, diffuse or infiltrative masses, such as mesothelioma or lymphoma, are difficult to identify via ultrasound in the early stages, often requiring correlation with other methods (cytology, biopsy, CT, etc.) [16].

**Figure 1:**
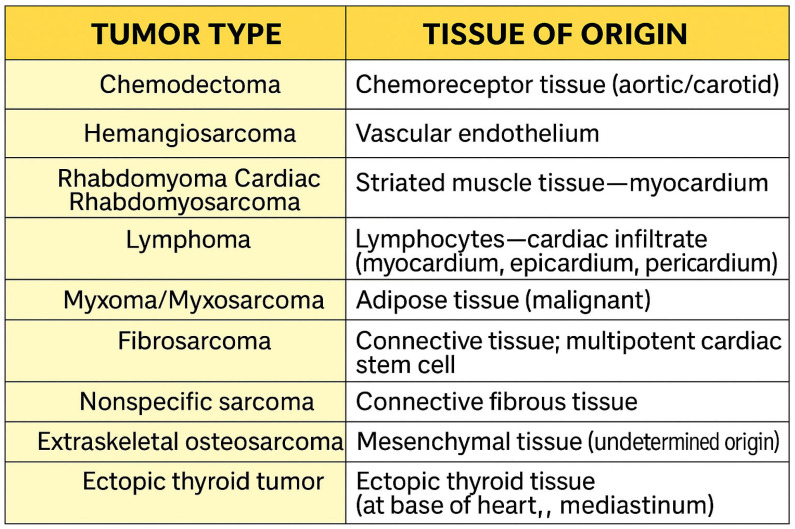
Histopathological classification of canine cardiac tumors by tissue of origin.

The diagnosis of cardiac tumor masses remains a clinical challenge, especially due to the variability in echocardiographic appearance and the difficulty of histological confirmation in vivo [29]. The classification of these tumors is essential for the therapeutic protocol. In practice, three types of classifications are predominantly used: oncological (benign vs. malignant; primary vs. secondary), histological (Figure 1), and topographic characteristics (depending on the location and echocardiographic appearance) [30]. Cardiac masses, classified into primary tumors (benign or malignant), secondary tumors (metastatic), and pseudo-tumors (such as thrombi, cysts, or valvular growths), require a correct diagnosis in order to establish an appropriate therapeutic plan; the imaging examination has an essential role in this, allowing morphological evaluation (size, shape, location, and mobility), tissue characterization (tumor composition: adipose tissue, necrosis, hemorrhage, and calcifications), the analysis of the involvement of adjacent structures (the invasion of the pericardium, large vessels or valves), and the determination of hemodynamic impact (the obstruction of blood flow or mass-induced valvular insufficiency) [29]. Most intracavitary tumors can be easily detected by transthoracic echocardiography (TTE), while transesophageal echocardiography (TEE) is indicated in cases where the acoustic window is limited, small masses are identified at the base of the heart, or invasion is observed into adjacent structures [31,32].

**Figure 2:**
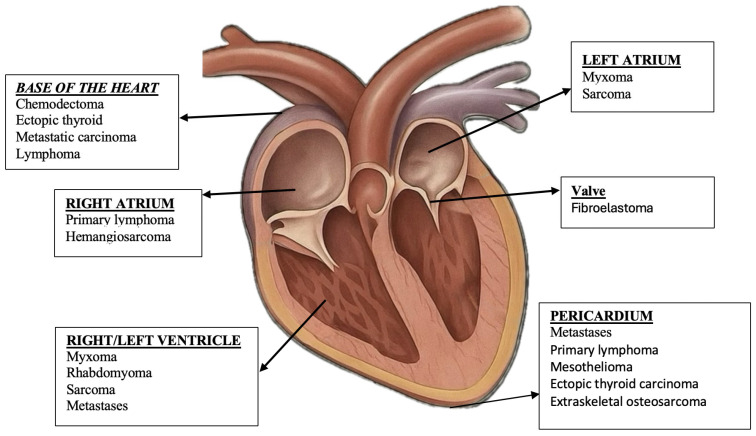
An overview of the location of cardiac tumors.

Cardiac tumors can originate from any compartment of the heart (Figure 2). However, certain histological types show a predilection for certain cardiac chambers. Thus, myxomas are located predominantly in the left atrium, lipomas are more frequently found in the right atrium or in the left ventricle, and fibromas and rhabdomyomas have a predominantly ventricular distribution [14,33]. Angiosarcomas are most commonly identified in the right atrium, while undifferentiated polymorphic sarcomas occur predominantly in the left atrium [34]. Although theoretically, they can affect any cardiac structure, the uneven distribution of these tumors justifies the adoption of a topographic approach centered on cardiac chambers in the process of differential diagnosis and imaging evaluations [35].

Recent studies indicate an increased prevalence of primary tumors, with hemangiosarcoma being the most commonly diagnosed type, especially in large breeds, such as Golden Retrievers and German Shepherds [1,36]. Its preferential location in the right atrium is particularly clinically relevant, as it is frequently involved in the occurrence of cardiac tamponade [31]. The prognosis remains reserved, with a short average survival after the onset of pericardial effusion, and current treatments (pericardiocentesis, chemotherapy, pericardiectomy) have not demonstrated significant improvements in lifespan [36,37].

### 3.2. Echocardiographic Diagnosis of Atrial Hemangiosarcoma

**Figure 3:**
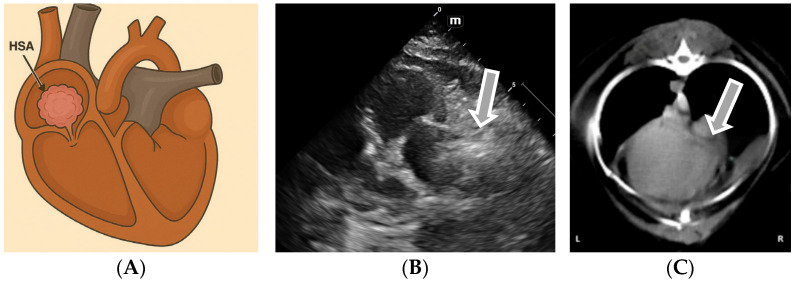
(**A**) The location of atrial hemangiosarcoma in canids; (**B**) a bidimensional (2D) echocardiogram (left apical 4 chambers view) in a dog with hemangiosarcoma invading the right atrial lateral wall; and (**C**) the tomographic appearance of an atrial hemangiosarcoma (original).

Hemangiosarcoma (HSA) is a highly malignant and aggressive tumor originating from the vascular or hematopoietic endothelium [38]. Cardiac hemangiosarcoma occurs primarily in the right atrial atrium and appendix or metastatic (Figure 3) when multifocal myocardial infiltrates are identified. Cases of diffuse infiltrative hemangiosarcoma involving only the atrial or ventricular wall are cited in the literature [20].

Hemangiosarcoma is frequently associated with pericardial effusions or cardiac tamponade, which are the consequence of blood vessel rupture [39]. Echocardiographic diagnosis has a sensitivity between 48% and 69% in the case of cardiac hemangiosarcomas [20]. The echocardiographic diagnosis of right atrial hemangiosarcoma is based on the identification of a heterogeneous mass, frequently associated with hypoechoic cavities, located in the right atrial appendix and/or right atrium [6].

In addition to qualitative assessments, the echocardiographic evaluation of cardiac hemangiosarcoma can be complemented by quantitative parameters. These include the measurement of mass dimensions (length, width, and area), their mobility during the cardiac cycle, and the assessment of chamber compression or collapse (e.g., right atrial or ventricular diastolic collapse), which are indirect signs of tamponade physiology [40,41]. Right atrial mass dimensions exceeding 2–3 cm in diameter are commonly reported, although variability is high due to tumor heterogeneity [42]. Two-dimensional (2D) echocardiography allows the morphological characteristics of the tumor to be highlighted, such as its extension in the pericardial space, contact with neighboring structures (aortic root and pulmonary artery), and the presence of a “floppy” external portion, rich in fibrin [43]. A transthoracic echocardiographic examination (TTE) is, in most cases, sufficient for preoperative evaluation, although TEE remains the reference method [43]. Optimal visualization of the lesion is obtained from the right long parasternal view, followed by probe adjustments to include the right atrial appendage, right atrium, tricuspid valve, and vena cava in a single plane. The slight inclination of the dorsal and cranial probe facilitates the assessment of the marbled texture of the mass and the determination of the degree of myocardial infiltration, which is essential for the therapeutic decision [44]. Additional echocardiographic techniques, such as Doppler imaging, can identify respiratory variations in ventricular filling, which are suggestive of cardiac tamponade. Tissue Doppler imaging (TDI) can detect early myocardial dysfunction even in the absence of gross structural abnormalities, while real-time three-dimensional echocardiography improves spatial resolution and provides a more accurate evaluation of tumor extension [45]. The right parasternal long-axis view is the primary window for detecting right atrial hemangiosarcoma. By adjusting the probe laterally and slightly counterclockwise, an off-axis view can be obtained that includes the right atrial appendage, right atrium, tricuspid valve, and venae cavae. Further dorsal and cranial angulation allows the tumor’s mottled texture and myocardial infiltration to be assessed [12]. When available, contrast-enhanced echocardiography (CEUS) improves diagnostic differentiation between neoplastic tissue and thrombus or necrotic areas based on the vascular enhancement pattern within the mass [46].

### 3.3. Echocardiographic Diagnosis of Chemodectoma

**Figure 4:**
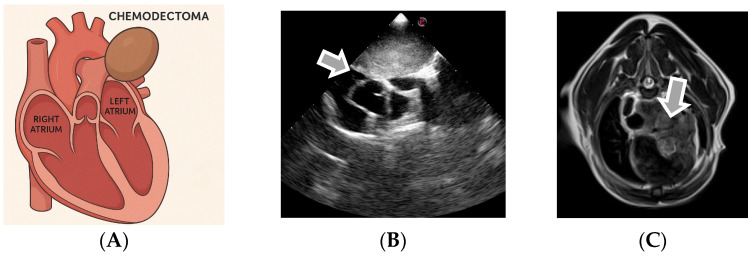
(**A**) The location of chemodectoma in canids; (**B**) a bidimensional (2D) echocardiogram (a parasternal short axis view of the base of the heart) in a dog with a heart base tumor; and (**C**) the magnetic resonance imaging features of chemodectoma in dogs.

Chemodectomas are primary cardiac tumors originating from chemoreceptor tissue, frequently located around the carotid bifurcation, the root of the aorta, the main pulmonary arteries, and the jugular veins (Figure 4) [12,47,48]. They present as nodular, soft, encapsulated, and homogeneous masses located predominantly at the base of the heart. Although they are generally solitary, they can occasionally be multiple and may be associated with other endocrine tumors [49]. Chemodectomas are usually locally invasive but can rarely metastasize to other parenchymas. In many cases, they are incidental findings during routine echocardiography and remain asymptomatic until the appearance of a pericardial effusion or signs caused by the compression of structures at the base of the heart [50]. The tumor can extend caudally to the left atrium, laterally, and to the right atrium and vena cava, as well as cranially around the pulmonary artery, sometimes surrounding its bifurcation. An echocardiographic evaluation of tumor extension is most commonly performed from the right long and short parasternal view but also from the left cranial parasternal view of the ascending aorta. The invasion of the left atrium and the possible compression of the pulmonary veins can be visualized from the left apical view with four cameras [12].

Chemodectomas are common in brachycephalic breeds and have benign characteristics but can become problematic due to the local invasion and compression of the basal structures of the heart. In advanced stages, ectopic thyroid chemodectoma and tumors may be echocardiographically indistinct, but they differ in growth patterns, with ectopic thyroid tumors extending along the ascending aorta and cranial mediastinal structures, unlike chemodectoma, which develops around the main pulmonary artery and its bifurcation [49]. For the correct evaluation and planning of the surgical approach, the use of transthoracic echocardiography (TTE) and transesophageal echocardiography (TEE) is recommended in order to identify the presence of mediastinal vessels inside the tumor mass [12]. In addition to the standard 2D echocardiographic evaluation, a comprehensive imaging approach should include the measurement of the tumor size, the delineation of its spatial relationship to the great vessels, and Doppler analysis to assess potential hemodynamic impact [51].

The quantitative assessment of chemodectomas should include measurements of maximal diameter, the cross-sectional area, and estimated volume, especially when planning surgical intervention. Masses typically range from 1.5 to 5 cm in diameter at the time of diagnosis, with larger lesions more likely to cause compressive signs [7]. On two-dimensional echocardiography, chemodectomas appear as homogenous, hypoechoic to mildly hyperechoic, and well-encapsulated masses. In some cases, internal vascularization can be assessed using color Doppler, though blood flow is often limited due to the compact structure of the tumor [52]. Doppler echocardiography may reveal altered pulmonary or systemic venous return in cases of significant vascular compression, particularly when the mass impinges on the caudal vena cava or pulmonary vein ostia. Tricuspid inflow velocities may also be affected by right atrial compression [51]. The optimal visualization of tumor extension can be achieved using a modified right parasternal long-axis view directed cranially to trace the mass along the ascending aorta and pulmonary artery bifurcation. The left cranial parasternal view enhances the assessment of the aortic root, while the subcostal view may help delineate contact with the vena cava [53]. In selected cases, when transthoracic echocardiography provides inconclusive data, advanced imaging modalities such as contrast-enhanced echocardiography (CEUS), computed tomography (CT), or cardiac MRI can further refine anatomical localization, evaluate vascular invasion, and distinguish chemodectomas from other mediastinal or heart base tumors with similar echocardiographic appearances [52].

This type of cardiac neoplasia is associated with slow growth and local expansion, but there have been studies showing a predisposition to metastasis in the lungs, spleen, and liver [14]. The echocardiographic assessment of tumor extension into the left atrium includes right parasternal long- and short-axis views, the left cranial parasternal view of the ascending aorta, and the left apical four-chamber view, which also reveals pulmonary vein compression [12].

Ectopic thyroid tumors arise from displaced thyroid tissue and can be located in the neck, cranial mediastinum, or at the heart base, reflecting their embryologic origin. When located at the heart base, small ectopic thyroid tumors often appear between the aorta and main pulmonary artery, making them initially indistinguishable from chemodectomas on echocardiography due to similar echotexture and location; the data are shown in Table 3. However, their growth pattern offers a key differentiating feature: ectopic thyroid tumors tend to extend dorsocranially, following the course of the ascending aorta, cranial mediastinal arteries, and cranial vena cava, whereas chemodectomas typically envelop the main pulmonary artery and its bifurcation. For accurate assessment and surgical planning, both TTE and TEE are recommended to identify mediastinal vessel involvement within the mass [12].

### 3.4. Echocardiographic Diagnosis in the Case of Other Cardiac and Pericardial Masses

Atrial myxoma, although rare, is an example of a benign tumor with a typical echocardiographic appearance, but it can be confused with malignant formations such as myxosarcomas. Atrial myxoma is rarely identified in dogs; they are benign masses that are located in the cardiac chambers. Echocardiographically, they are uniform tissue structures with smooth edges; thus, differential ultrasound diagnosis should be made with paraganglioma. Myxosarcomas are malignant, highly infiltrative endocardial structures of mesenchymal origin, which can affect the pulmonary artery, the right ventricle and atrium, the left ventricle, and the pericardial sac. Echocardiographically, they have a multilobed and heterogeneous appearance, containing small hypoechoic areas. The ultrasound differentiation of benign atrial myxoma from myxosarcomas is difficult despite the small existing peculiarities [20].

Primary intracardiac tumors develop from the myocardial wall and can be difficult to differentiate echocardiographically from intracardiac thrombi [12,47]. Thrombi usually occur in the context of coagulation disorders and are located in areas with low blood flow, such as the right atrium or the main pulmonary artery. A useful sign is the presence of a thin anechoic margin between the endocardium and thrombus [16].

**Figure 5:**
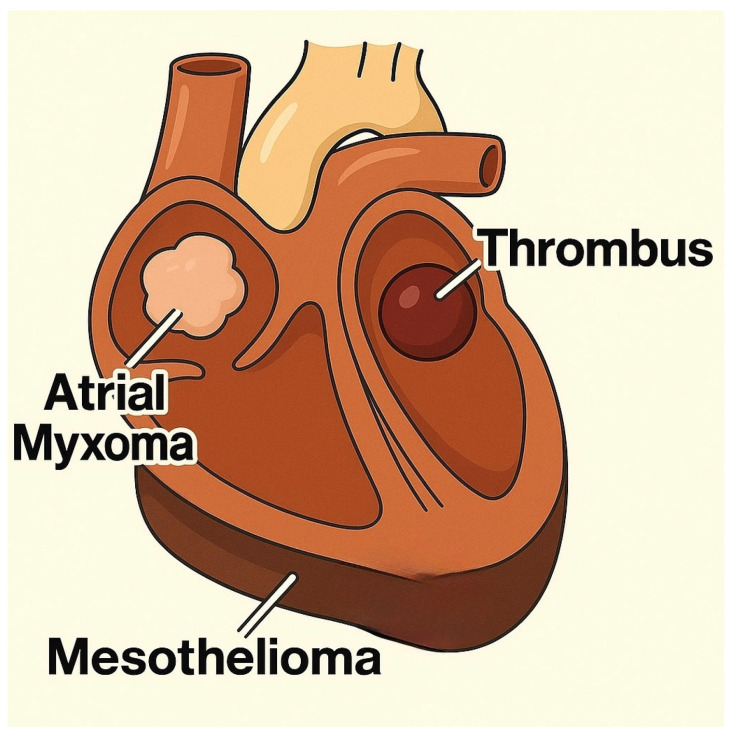
The other possible locations of cardiac/pericardial tumors.

Metastatic tumors are relatively common and can reach the heart through anatomical continuity, the lymphatic system, the caudal vena cava, or the coronary arteries (Figure 5) [34]. They are especially noticeable in the free wall of the left ventricle. Metastatic tumors (HSA, lymphoma, melanoma, and carcinoma) can appear as intramural disseminated nodules with a heterogeneous texture and lack of systolic contraction in the invaded segments [49]. Epicardial metastases can cause acute pericardial tamponade via rupture.

Mesothelioma and lymphoma are neoplastic entities that are difficult to diagnose via ultrasound due to their diffuse appearance and lack of an organized mass [12,49]. In these cases, chronic or hemorrhagic pericardial effusion may be the only clinical clue, justifying further diagnostic explorations [47]. Mesothelioma is a rare, diffuse malignant tumor that causes intracavitary hemorrhagic effusions, especially in elderly dogs and cats. The juvenile form has been reported in isolated cases [34]. The tumor derives from mesodermal cells of the pleura, peritoneum, pericardium, or testicular vaginal tunic. In the early stages of the disease, mesothelioma is not visible on ultrasound or CT due to a lack of tumor organization. In advanced stages, it can form polyps or nodules, affecting the entire serum. Distant metastases are rare. In males, especially German Shepherds, a sclerosing form with fibrous thickening of the pleura or peritoneum may occur [34].

Lymphoma is a cardiac neoplasia identified more often in cattle and felines, but it is also occasionally reported in dogs [16,20]. It is characterized as a type of infiltrative neoplasia, which can affect the heart muscles or pericardium. Cardiac musculature is described echocardiographically as hypokinetic and hypertrophied, with or without areas of increased echogenicity, and may be associated with pericardial effusion. Pericardial lymphoma is associated with cardiac tamponade without the presence of echocardiographically visible structural changes [20].

### 3.5. Therapeutic Protocols

Therapeutic protocols in the case of cardiac neoplasms aim, in the first instance, at the clinical stabilization of the patient, given that clinical manifestations can range from subclinical forms to acute life-threatening syndromes, regardless of the histological type of the tumor [11]. The symptomatology is most often secondary to cardiovascular dysfunction or fluid accumulation in the pericardial cavity [53,54]. In these contexts, emergency management focuses on controlling the tumor hemorrhage [55], stabilizing heart rhythms, and diminishing the compressive effects exerted by tumor formation on intracardiac structures.

The determination of the pH of the pericardial fluid has proven to be a useful tool in differentiating pericardial effusions of neoplastic and non-neoplastic origin, thus contributing to the orientation of the differential diagnosis and to the outlining of an appropriate therapeutic approach [56,57].

Because primary cardiac tumors are difficult to address surgically, and the definitive histological diagnosis involves cytological or biopsy sampling—procedures that are often inaccessible or risky in their current practice—the therapeutic approach is frequently guided by the echocardiographic aspect [57]. Depending on the type and location of the lesion, treatment may include methods such as pericardiocentesis, surgery, chemotherapy, or radiation therapy [58,59]. Surgery is rarely curative, given the proximity of tumors to large vessels and vital structures, but it can contribute significantly to improving symptoms and prolonging life span [60].

For cardiac lymphoma, the effectiveness of combined chemotherapy protocols has been demonstrated, while in the case of hemangiosarcoma, adjuvant chemotherapy is recommended, although the prognosis remains reserved [61]. Mesothelioma tumors or other diffuse neoplasms are difficult to diagnose echocardiographically due to the lack of a well-defined mass, which is why chronic or hemorrhagic pericardial effusion may be the only clinical indication requiring advanced diagnostic explorations.

Monitoring the evolution of these patients through repeated echocardiography at intervals of 3–6 months is recommended to evaluate tumor progression and adjust therapeutic strategies. The current protocols are mainly focused on cardiac tumors with high incidence, such as hemangiosarcoma and chemodectoma. In this regard, toceranib phosphate (Palladia), administered orally at home, is used for the treatment of chemodectoma [62], and for cardiac hemangiosarcoma, doxorubicin is recommended, administered intravenously at intervals of 2–3 weeks [63]. Currently, further research is needed to optimize the therapeutic management of other cardiac tumor entities.

## 4. Conclusions

Cardiac tumors in dogs, although initially considered rare entities in veterinary medicine, register an increasing incidence, which is a phenomenon closely correlated with the development of imaging technologies, especially echocardiography and advanced techniques such as CT and MRI with contrast agents. The early identification of these formations—primary or secondary and benign or malignant—remains essential in establishing an effective prognosis and therapeutic plan. Right atrial hemangiosarcoma is the most common form associated with pericardial effusion and cardiac tamponade, and chemodectoma is distinguished by its basal location and association with brachycephalic races. Echocardiographic diagnosis, with a variable sensitivity depending on the tumor type, allows for a precise morphological and functional evaluation complemented by advanced investigations and, in some cases, biopsy or cytology. Clinical manifestations are often non-specific, requiring an integrated clinical-paraclinical approach. Therapeutic management, although limited in curative efficacy, has seen significant advances, including pericardiocentesis, oncological treatments (chemotherapy and toceranib), and palliative interventions, depending on the histological type, location, and evolutionary stage. In conclusion, cardiac tumors in dogs remain a major challenge for clinicians, and the success of the approach depends on early recognition, multiple integrations of diagnostic methods, and the constant updating of therapeutic protocols.

## Figures and Tables

**Table 1:** Auscultation findings in canine cardiac tumors.

Tumor Type	Auscultation Findings
Hemangiosarcoma	– Low-intensity murmurs may occur due to altered blood flow; – Muffled heart sounds are common with pericardial effusion or tamponade.
Chemodectoma/Aortic-body Tumor	– Murmurs uncommon unless causing significant pericardial effusion or compression; – Heart sounds often muffled with large effusions.
Lymphoma (cardiac infiltration)	– Murmurs typically absent or soft; – Heart sounds may be diminished if effusion develops; – Gallop rhythms possible with restrictive physiology.
Fibrosarcoma/Undifferentiated Sarcoma	– Sparse auscultation data; – Soft to muffled sounds may occur with effusion or chamber involvement.
Extraskeletal Osteosarcoma	– No specific murmurs described; – Muffled heart sounds possible if pericardial infiltration or effusion is present.
Mesothelioma	– Soft or muffled heart sounds due to extensive pericardial effusion; – Gallop rhythms may occur if pericardial constriction develops.
Ectopic Thyroid Tumor	– Murmurs unlikely unless there is outflow obstruction; – Muffled sounds possible with large mediastinal mass or effusion.
Myxoma/Myxosarcoma	– Rare; – Soft or low-grade murmurs may occur with valvular interference; – Muffled sounds possible with effusion.

**Table 2:** Clinical signs according to primary cardiac tumor types in dogs.

Tumor Type	Common Location	Main Clinical Signs
Hemangiosarcoma	Right atrium, pericardium	Lethargy, exertional syncope, jugular distension, ascites, collapse, dyspnea
Chemodectoma (Paraganglioma)	Heart base (aortic bifurcation)	Chronic cough, dyspnea, bradycardia, exercise intolerance, tracheal compression
Lymphoma	Myocardium, pericardium, atria	Arrhythmias, tachycardia, lethargy, congestive heart failure, generalized lymphadenopathy
Mesothelioma	Pericardium	Dyspnea, gallop rhythm, pericardial effusion, cough, signs of right-sided heart failure
Myxomas/Myxosarcomas	Cardiac valves, chambers	Heart murmur, signs of obstruction, mild dyspnea, reduced exercise tolerance
Ectopic thyroid tumors	Heart base/anterior mediastinum	Heart murmur, mediastinal compression, dyspnea, cough

**Table 3:** Comparative table—differential diagnosis of chemodectoma vs. ectopic thyroid tumors in dogs.

Feature	Chemodectoma	Ectopic Thyroid Tumor
Common Location	The base of the heart, near aortic root and pulmonary artery	Cranial mediastinum, near the base of the heart
Vascular Involvement	Encircles pulmonary arteries and may compress atria	Surrounds great vessels (cranial vena cava, aorta)
Echogenicity	Homogeneous, hyperechoic	Homogeneous, hyperechoic
Mobility on Echo	Non-mobile	Non-mobile
Contour	Smooth margins	Smooth margins
Growth Speed	Very slow	Slow
Infiltration	Extremely rare	None
Number of Masses	Typically single	Single
Other Clues	Often incidental and predisposed to brachycephalic breeds	May be functional (thyroid hormones)
